# Mental Health Care Access Among Arab Immigrants in the United States: Application of Public Arena Model

**DOI:** 10.1007/s10597-023-01182-2

**Published:** 2023-10-26

**Authors:** Elrefaay Shaimaa, Stella Bialous

**Affiliations:** 1 School of Nursing, UCSF, 2 Koret Way Rm 411Y, San Francisco, CA 94143 USA; 2 School of Nursing, Tanta University, ElGeish Street, Gharbiya, 31257 Egypt; 3 Department of Social and Behavioral Sciences, UCSF School of Nursing, San Francisco, CA 94143 USA

**Keywords:** Arab immigrants, Accessibility, Public arena model, Mental health

## Abstract

Arab immigrants constitute a sizable portion of the US population, and their adjustment and relocation challenges might escalate mental health issues. Nevertheless, mental health care accessibility among such populations is not recognized as policy issue. Hence, it is crucial to explore the political tools that might be employed to improve immigrants’ access to mental health treatment. The Public Arenas Model (Hilgartner and Bosk, American Journal of Sociology 94:53–78, 1988) provides better understanding of how access to mental health care is defined in the public sphere, why mental health inaccessibility among Arabs has not received attention, and how stakeholders worked to raise the public’s attention to such issue (Smith, Policy, Politics & Nursing Practice 10:134–142, 2009). Ultimately, several policy options are proposed to address Arab immigrants’ access to mental health care issue, including increasing mental health service providers’ language proficiency and cultural competency, integration of behavioral health and primary care services for immigrant populations, and considering novel modes of mental health delivery.

## Introduction

Immigrants account for a sizable proportion of the United States (U.S.) population, and immigration into the U.S. is expected to continue to increase until 2050 (Ikonte et al., 2020). In 2019, the U.S. sheltered around 44.9 million immigrants, representing approximately 13.7% of the total population (Zong, Batalova & Hallock, 2019) and immigration is expected to continue to increase until 2050 (Ikonte et al., 2020).Between 2000 and 2019, Arab population in the U.S. doubled from 596,000 to 1.2 million, and about 82% of whom were citizens of the U.S (Albqoor et al., 2020; Albqoor et al., 2021; US department of Homeland Security, 2020). Arab immigrants may endure multiple difficulties after their relocation to the U.S., including physical separation from their family, obstacles both cultural and linguistic, and adjustment to a new, frequently hostile, environment (Alkaid et al., 2021; Suleiman et al., 2021). These difficulties and the accompanying stresses can and may aggravate pre-existing mental health conditions or provoke new mental health problems such as anxiety, depression, or other traumatic stress disorders, to name just a few (Bulut & Brewster, 2021; Derr, 2016; Ikonte et al., 2020).

However, Arab immigrants remain underrepresented in the mental health care system, despite the need for care they might be expected to have, based on the frequency of pre-existing mental health conditions and their adaptational stresses (Bulut & Brewster, 2021; Derr, 2016). Arab immigrants experience greater mental health challenges and tend to use mental health services at lower rate than their cultural equivalents (Ayele, 2020; Pampati et al., 2018). For example, according to the National Health Interview Survey, foreign-born individuals from the Middle East report greater odd for serious emotional distress than American-born non-Hispanic Whites (Papmti et al., 2018). Moreover, Abuelezam et al., 2019 in their recent systematic review indicated that Arab Americans had higher prevalence of no insurance and living below the federal poverty level than non-Hispanic Whites. Arab immigrants tend to shun specialized mental health care due to structural barriers as well as the residual cultural, negative, perceptions they may share of such services (Ayele, 2020; Elshahat, Sarah & Moffat, 2021) The most frequently cited structural barriers include the cost of care, lack of health insurance, lack of access to transportation, language barriers, encounters with discrimination, their economic position, and insufficient access to the health care system (Ayele, 2020; Khan et al., 2019). The effect of untreated mental conditions extends beyond an individual’s or their family’s suffering (Ayele, 2020). Ignoring mental health disorders can result in undue disability, unemployment, poverty, drug dependence, homelessness, improper incarceration, suicides, and poor quality of life (Segal et al., 2020). More significantly, according to the National Alliance on Mental Health, untreated mental illness costs the U.S. up to $300 billion in lost productivity each year which affects national economic development (Hunt et al., 2015; Sherr, 2021). Therefore, it is critically important to explore the policy tools that might be employed to improve Arab immigrants’ access to mental health treatment.

Although earlier research has indicated inaccessibility to such care among Arab immigrants as a clinical issue (Abo-Rass, et al., 2022; Khan et al., 2019) this inaccessibility is not recognized as a social or policy concern. To focus public attention on this issue, it must be re-contextualized (Khan et al., 2019). Classifying access to culturally competent mental health care as a community problem suggests that the need for such care may be greater than the mere perceived mental health impediments of the specific Arab immigrant population (Smith, 2009). The Public Arenas Model (1988) enables more thorough examination of the definition of access to mental health care in the public sphere, how competition with other clinical issues has influenced the framing of this specific issue, and how various operatives continue to influence the perception of mental health care approachability as well as the impact of such care on the Arab immigrant population (Smith, 2009).

The aim of this paper is to introduce the Public Arenas Model’s central elements and analyze its applicability as a framework for understanding the policy issue of the underrepresentation of Arab immigrants in the mental health care system. Specifically, the paper will explore (1) how the Arab minority’s underutilization of mental care has been previously defined at least imprecisely as an issue of cultural and language incompetency instead of the more accurate assessment as a lack of access due to economic burden and the cost of mental care, (2) how the efforts of many stakeholders to advocate, inform, and influence the public’s attention to mental health care in the Arab minority has strengthened this issue as a prominent topic of public debate, and (3) proposing some policy solution to address the issues of access to mental health care for Arab immigrants. The outputs of the public Arena model will be crucial for parties involved in the mental health convenience problem, and the nursing profession will be better able to influence health policy by defining Arab immigrants’ representation in mental health services (Smith, 2009). Using the framework of the public arena in research may help nurses design new research questions that address Arab population inaccessibility to mental health facilities (Smith, 2009). Nurses also may be better able to influence health policy if they understand how mental health issues compete with other health issues for public attention.

## A Brief Background of Arabs’ Mental Health Service Utilization

Racial and ethnic minorities’ disparate mental health outcomes have been the subject of increased concern among mental health professionals and researchers since the 1980s (Perzichill, 2020). Discrepancies in access to mental health services, usage patterns, and treatment outcomes among racial and ethnic minorities have been acknowledged (Olfson et al., 2023; National Institute of Mental Health). Evidence from earlier research suggests that Arab minorities are considerably less likely to need or receive mental-health services than Whites (Abuelezem et al., 2019; Elshahat, Sarah & Moffat, 2021) Cultural experiences and religiosity may theoretically act as a shield against emotional concerns for cultural and ethnic subgroups; for example, religion has conventionally been recognized as a major defense mechanism for Arab Americans (Alkaid Albqoor et al., 2021; Hall, 2020). According to an online Survey of Arab Immigrants, intended to ascertain the identification of, and response to, distressing symptoms by Arab immigrants, most Arab immigrants did not seek mental health care in response to emotional illness (Neighbors et al., 2007). Lasser and colleagues (2002) also discovered that, in both primary care and psychiatric settings, Hispanic/Latinos and Arab minorities had lower rates of visits for psychotherapy, pharmacotherapy, and psychological counseling than Whites. These gaps may exist due to one or more of the following: (1) a lack of trust between clients and health care workers; (2) language and cultural hurdles; (3) treatment and custody issues for racial and ethnic minorities; (4) a greater frequency of diagnoses of anxiety or depression in Arabs; (5) an absence of health insurance coverage; and (6) clinical prejudice among nonminority facility contributors (Alshamsi et al., 2020; Olfaon et al., 2023; Priester et al., 2016; Tahir et al., 2022). Amer and Hovey (2007), who examined disparities in acculturation and service consumption among 120 first- and second-generation Arab Americans, discovered that Arab females and married Arab immigrants reported a stronger sense of Arab ethnic identification. Due to strong cultural links, many Arabs avoid psychological therapies, which are more intrusive than physical services (Amer and Hovey, 2007). Researchers have discovered that an individual’s stance regarding obtaining “formal mental health services” can significantly impact their choice to seek professional help when signs of mental health problems manifest (Derr, 2016; Ikonte et al., 2020; Khan et al., 2019). The relationship between an individual’s views and subsequent conduct can provide critical insights into cultural variation in help-seeking behaviors (Abo-Rass, et al., 2022; Bulut & Brewster, 2021).

## Stakeholders

Stakeholders are entities, participants, or parties that have an interest or a concern in a dispute. How policymakers address policy challenges directly impact stakeholders (Smith, 2009). Many organizations have conferred interests in or are involved or influenced by the consequences of their choices concerning the underrepresentation of Arab immigrants in mental health treatment (Abuelezam et al., 2018; Tahir et al., 2022) Arab minority individuals and their families are conceivably the most significant stakeholders, but they possess little actual influence or authority over the issue. They may attempt to obtain relative authority over their access to health care services, but they face the same impediments to care previously discussed (e.g., limited transportation access, limited English proficiency, and a lack of health insurance) (Derr et al., 2016) Groups or organizations providing support to immigrants, such as the Immigrant Legal Resource Center (ILRC), Migrant Health Promotion, and the National Alliance for Immigrants, are more powerful stakeholders than Arab individuals. These organizations advocate for migrants and have a greater bargaining ability with politicians due to their accessible assets (Eldeeb & Sherief, 2017; Almutairi et al., 2022). Mental health care professionals are also stakeholders. Although they can assist by providing mental health care to immigrants or at the very least to underline their relevance to other stakeholders, they are also constrained by resource limits (Eldeeb & Sherief, 2017; Tahi et al., 2022).

The Health Resources and Service Administration, a division of the United States Department of Health and Human Services, is another stakeholder as it is responsible for overseeing government-funded immigrant health facilities. This institution wields considerable influence and control over the health care supplied to immigrant groups but runs on a tight budget and is tasked with resolving often clashing concerns, including homelessness, public housing, and health care (U.S. Department of Health and Human Services, 2020). These stakeholders and the venues in which they operate contribute to the capacity of the policy issue of access to mental health treatment for Arab immigrants as a significant social concern (Smith, 2009).

## Hilgartner and Bosk’s Public Arena Model

Hilgartner and Bosk’s (1988) Public Arenas Model, aims to explain the reasons for the classification of a selective group of emergent social circumstances over others (despite their competition for public recognition) as issues in distinct arenas, can be utilized as a framework to understand the policy issue of underutilization of mental health care among U.S. Arab immigrants (Hilgartner & Bosk, 1988). According to Hilgartner and Bosk (1988, p. 55), a social issue is “a putative condition or situation which is labeled as a problem in the arenas of public discourse and action”; they propose that the objective value of the condition, regardless of its magnitude, has no bearing on whether it is classified as a social issue, but rather that public consideration is a limited resource allotted via a competition existing within a network of public venues (Hilgartner & Bosk, 1988).

### Key Concepts in the Public Arenas Model

There are six central concepts in the Public Arenas Model: (1) a dynamic rivalry of social issues, (2) the institutional or public arenas, (3) the carrying capacity of each arena, (4) the selection criteria for problem survival, (5) the interface or synergy between arenas, and (6) an arena’s operators (Hilgartner & Bosk, 1988). According to Hilgartner and Bosk, *societal issues compete* on two levels. First, within each substantive domain, multiple framings of the issue may contend for authority. Second, a significant number of public issues vie for public attention (Hilgartner & Bosk, 1988). Social problems and the operators who endorse them must compete to be in and endure on the public schema (Hilgartner & Bosk, 1988). Furthermore, no substantial correlation exists between social issues’ success or failure in this competition and the number of persons impacted the amount of harm (as judged by any established criteria) or any other neutral variables that pretend to evaluate significance (Hilgartner & Bosk, 1988).

According to Hilgartner and Bosk (1988), the second concept is the *public or social arena* in which social issues are debated, selected, defined, and presented to the community. These venues may include governmental authorities, courts of law, media outlets (television, journals, broadcasts, theatrical films, and news outlets), religious groups, political administrations, and novels that describe social concerns (Hilgartner & Bosk, 1988). Hilgartner and Bosk use the term *“carrying capacity”* to refer to the number of social problems that a given public venue (e.g., the media outlet television) can address at a given time. Thus, although the number of potential problems is enormous, the carrying capacities of the available venues impose severe limits on public presentations of problems (Hilgartner & Bosk, 1988; Smith, 2009). Carrying capacity is quantifiable in a variety of ways, both institutional and personal, including (for institutions) time (e.g., minutes of airtime, for television), space, personnel, resources, and money, as well as (for individuals) human characteristics such as the amount of energy, devotion, or compassion which an issue may command (Hilgartner & Bosk, 1988; Smith, 2009).

Hilgartner and Bosk (1988) established a set of *selection principles* shared by all public venues that effectively control social issues’ competitive process. These selection principles operate differently depending on the public venue under consideration (Hilgartner & Bosk, 1988). They include the intense competition for prime space, a requirement for drama and novelty, a possible problem with saturation, the rhythm of organizational life, cultural (or societal) preoccupations and political biases (Hilgartner & Bosk, 1988). Each of these principles are governed by the venue’s carrying capacity, i.e., if the capacity is small, the competition will be more intense, more drama and novelty may be required to catch the public’s interest, saturation (both positive—the initial introduction of the issue—and negative—when the public perceives too much of the issue) will be easier to achieve, the organizational rhythm will be faster, and the last two principles (cultural preoccupations, political biases) may be more acutely brought to bear on the issue (Hilgartner & Bosk, 1988). Hilgartner and Bosk’s (1988) feedback describes the complex web of connections and actions existing between venues (arenas) which may either magnify or diminish the attention paid to problems in that arena. Players’ activities inside these arenas impact the feedback mechanisms and other aspects of the competition between issues (Hilgartner & Bosk, 1988). These players, or *operators*, maybe people such as elected politicians, policy experts, or community planners, or they may comprise entities such as alliances or distinct interest assemblies (Hilgartner & Bosk, 1988). Operatives build networks on shared interests to advance their respective issues (Hilgartner & Bosk, 1988).

## Application of the Public Arenas Model to Arabs’ Underrepresentation in Mental Health Care

There is competition to the perception of Arabs’ accessibility to mental health treatments as a problem. Daily, the public is bombarded with an assortment of societal concerns using methods designed to capture their attention. According to Hilgartner and Bosk (1988), assertions about societal problems do more than draw attention to specific circumstances; they also “scaffold” them in a certain way (Hilgartner & Bosk, 1988).

The characterization and mental constructs around mental health care unapproachability vary among Arabs (as well as the public) according to the arena in which it is debated, and which operatives are engaged (Hilgartner & Bosk, 1988).

Within the health arena, the issue of mental health care among Arab minorities is often defined as a problem of incompetency rather than an issue of mental health or allocation of resources; such incompetency may be perceived to include cultural incompetency, linguistic issues, mental health care disparity, or a lack of education or awareness among Arab groups about their own mental health needs (Moffat, 2021) in addition to discriminatory experiences (Schouler-Ocak et al., 2016). Thus, such prejudice and cultural difficulties affecting Arabs’ attitudes and hindering from seeking mental health care services (Elshahat, Sarah & Moffat, 2021; Schouler-Ocak et al., 2016). Thomson et al. (2015) state that the problem of Arabs’ inaccessibility to mental health care may also be defined (instead of structural impediments) as the lack of access to mental health care services due to a lack of knowledge of such services as well as a lack of awareness of their own mental health needs (Thomoson et al., 2015). Thomson et al.’s (2015) definition bolsters the health arena’s claim for culturally appropriate care and an education campaign intended to increase an individual’s access to mental health information.

The political arena, specifically the administrative and legislative divisions of authority, delineates the underrepresentation of Arabs in mental health care because of the interaction of (1) uncoordinated health care planning, (2) an insufficient number of mental health personnel, (3) inadequate health insurance coverage, and (4) remote mental health services (Derr, 2016; Tahir et al., 2022). The cultural venue may explain the inaccessibility of mental health services due to the societal stigma sometimes attached to mental health care (Dardas, Simmons, 2015). Each of these problem definitions represents a distinct view of mental health care services (Hilgartner & Bosk, 1988). (Hilgartner & Bosk, 1988). According to Hilgartner and Bosk (1988), the definition (and sometimes the venue) that eventually prevails in the related public discourse has substantial consequences for the social problem’s future development, the stakeholders concerned, and policy development (Hilgartner & Bosk, 1988). Although competition between venues as well as different portrayals of the same social issue will end with the decision regarding which portrayal will become the authoritative description, the broader rivalry also works to progress the issue of mental health inaccessibility among Arabs as a public health issue in general (Derr, 2016; Smith, 2009).

Carrying capacity, feedback across arenas, and operational links are all components of the Public Arenas Model, enabling the issue of Arabs’ mental health inaccessibility to gain traction (Hilgartner & Bosk, 1988). Carrying capacity components are unique to each public venue (Hilgartner & Bosk, 1988). Although there are structured and active groups representing the concerns of various Arab immigrant communities, the Arab minority constitutes a numerically small and highly marginalized community in the United States (Ikonte et al., 2020; Khan et al., 2019). As a result, the carrying capacity of these public venues is constrained (Khan et al., 2019). However, drawing the public’s attention to the financial inaccessibility of mental health treatment may provide a selection principle that might assist the overall inaccessibility issue to compete successfully with other issues for public attention (Hilgartner & Bosk, 1988). According to Hilgartner and Bosk (1988), “economic developments can affect communal conceptions of social problems” (p. 64). The present economic predicament due to the COVID19 pandemic and the health care burden are two of the significant concerns dominating the public conversation, which provides a lens to examine the financial implications of mental health care inaccessibility (Khan et al., 2019; Sher et al., 2021). Untreated mental illness costs the U.S. up to $300 billion in lost productivity each year (Sher et al., 2021). Combining Arab underrepresentation in mental health services with these cultural challenges might provide greater traction to the overall issue of Arab immigrant mental health care inaccessibility than that issue would have on its own (Elshahat, Sarah, and Tina Moffat, 2021; Hilgartner & Bosk, 1988; Khan et al., 2019).

Drama and novelty are other criteria that impact the identification as an issue of Arab’s approachability to mental health care (Hilgartner & Bosk, 1988). Popular culture has contributed to the drama and novelty surrounding mental health care issues (Ikonte et al., 2020; Smith, 2009). Stories about the mental health needs of immigrants and their children, as well as the health consequences of untreated mental illness, are theatrical (Smith, 2009). Dramatic stories implying that untreated mental illness contributes to significant issues such as depression and suicide risk help draw attention and spur public speech concerning the rising rates of mental health issues among the Arab minority and focus the public’s attention on targeted policies (Ikonte et al., 2020; Smith, 2009).

Within the context of Arab mental health care accessibility, culture acts as the most pervasive selection principle (Bulut & Brewster, 2021). According to Hilgartner and Bosk (1988), some issue categories are inextricably linked to broader societal concerns (Hilgartner & Bosk, 1988). Culture has been proven to influence the characterization by Arab immigrants of their emotional symptoms, the amount of support they receive from family and community, and how they cope with and seek treatment for mental illness (Ikonte et al., 2020; Tahi et al., 2022). For example, in Middle Eastern cultures, the traditional assumption that seeking mental health care will bring shame and disgrace to the family causes some people to internalize their symptoms rather than seek counseling (Olfaon et al., 2023; Priester et al., 2016). Arab Americans have been demonstrated to be more inclined than Whites to deal with personal difficulties and discomfort independently or seek assistance from their faith (Ayele, 2020; Derr, 2016). In terms of social support, researchers discovered that some Arab immigrants choose to seek assistance from other family members and members of their religious community rather than seeking mental health care (Ikonte et al., 2020; Tahi et al., 2022). Thus, framing the issue most relevant to society’s concerns and interests is critically important to achieve the reclassification of Arabs’ inaccessibility to mental health services as a social problem. This culturally sourced selection factor also has consequences for the deterioration in the portrayal of Arab mental health care as a societal concern.

## Proposed Policy Interventions

One policy option to address the issues of access to mental health care for Arab immigrants is to increase language access and improve the cultural competency of service providers. Cultural competence has emerged as a foundation for social work education and practices in pluralistic societies such as the U.S. and is considered an essential element in promoting effective mental health services to all populations (Tan, Allen, 2021). The Office of Minority Health of the U.S. Department of Health and Human Services has developed standards for culturally and linguistically appropriate services (Ohta, 2015). These standards focus on delivering culturally competent care, staff diversity, the need for ongoing education and training, language assistance services for those with limited English proficiency, and a strategic plan that addresses goals and accountability (Ohta, 2015). The National Alliance on Mental Illness (2012), an advocacy organization, recommends that all agencies receiving federal assistance should fully understand and comply with their obligations to provide good quality and equal treatment.

The possibility of obtaining such treatment helps patients to feel more comfortable with their provider and may aid them to continue to seek out mental health care (Phillips, 2020). Another significant advantage of this approach is tailoring mental health care to the recipients, which helps to ensure the appropriateness of the treatment for those individuals and enable culturally grounded coping strategies (Alshams et al. 2022; Phillips, 2020). In Fernández-Gutiérrez et al., 2018 systematic review, it was determined that cultural competence interventions targeted at immigrant groups can greatly enhance their functional health literacy. Alshams et al. 2022 stated that patients who speak the local language will be more satisfied with their healthcare and have greater utilization of essential sources of healthcare. However, extensive time and funding are required to develop the infrastructure necessary to ensure cultural competence training plans, linguistically relevant materials, recruit appropriate linguistic interns and employees, and adopt and adapt diverse staff recruitment programs (National Alliance on Mental Illness, 2012; Phillips, 2020). Moreover, cultural competence demands an ongoing commitment and multipronged approach; mental health care organizations must build an infrastructure that supports the activities and protocols to ensure culturally competent mental health practices (Grandpierr et al., 2018). Finally, organizations must weather pushback from staff members who may already consider themselves culturally competent because they know cultural competence as it applies to racial and ethnic diversity (Grandpierr et al., 2018; Phillips, 2020; Zolezzi et al., 2018).

Other, less ideal policy options exist. It comprises advocating for integrating behavioral health and primary care services for immigrant populations (Falconer et al, 2018; Peer et al., 2023). This approach entails central components, including patient-targeted alignment, inclusive and harmonized care, permanent access, and a system-based tactic (Odgkinson et al., 2017). Researchers have demonstrated that incorporating mental health services into primary care enhances both physical and mental health outcomes, in part because of integrating linguistic services on the provision of care to immigrants with complicated health care issues (Aupont et al., 2013; Kolk et al., 2014). Integration may also help Arab immigrants with severe emotional disturbances reduce their symptoms while enhancing retention and access to care (Odgkinson et al., 2017; Peer et al., 2023), possibly by reducing the associated stigma via more accessible treatments in a trusted environment (Hodgkinson et al., 2017). Evidence from prior literature indicates that that an integrated behavioral care approach has the possibility of promoting engagement with mental health services and maintain care continuity, while decreasing costs of care and stereotyping associated with behavioral healthcare (Falconer et al, 2018; Peer et al., 2023). However, this policy approach faces significant obstacles, as it requires enough mental health clinicians and support workers to effectively facilitate cooperation with physical medical specialists; clinics in larger cities may be better positioned to provide such integrated services (Odgkinson et al., 2017; Knopf et al., 2016). Facility-imposed time restrictions on physicians may hinder ensuring that good quality mental health treatments are provided, i.e., like those of a mental health professional (Knopf et al., 2016; Peer et al, 2023). Primary care physicians also lack the necessary training and support to confidently identify patients with significant mental health issues (Knopf et al., 2016).

A third policy option is to explore suitable novel and creative modes of service delivery (Sunjaya, 2020). E-mental health therapies increase access by providing advanced services along with support alternatives for individuals (i.e., immigrants) suffering mental illnesses (Sunjaya, 2020; Ashfaq et al., 2020). Such e-mental services may provide services more effectively to underserved immigrant populations (especially those wishing to avoid stigma, who prefer anonymity, or who cannot abide the clinical setting) and isolated residents (e.g., rural individuals) who cannot currently obtain services using the traditional approach (Liem et al., 2021). Many developing technologies (e.g., videoconferencing via Skype or Zoom) are now more prevalent and could be employed as online-based therapy to help shape future service interactions (Sunjaya, 2020; Ashfaq et al., 2020). A greater reliance on e-mental health solutions will benefit both clients and clinicians (liem et al., 2021).

Consideration and incorporation of the potentially acceptable electronic solutions into future service delivery will enable organizations to handle the provision of mental health care to Arab immigrants (Sunjaya, 2020; Ashfaq et al., 2020). Ashfaq et al., 2020 systematic findings shows the utility of technologies as screening methods for Syrian and Arab refugees. Hassan and Sharif (2019) provide evidence that telepsychiatry programs are more effective than conventional mental health therapy for refugees. However, tremendous effort and resources are necessary to develop the required training programs to provide mental health services (Cowan et al., 2019). To support the growth of such services, applicable legislation must be revised to effectively accommodate the new forms of treatment (Cowan et al., 2019). Finally, such services’ long-term sustainability may pose a concern if limitations are imposed on their cost, service reimbursement, or a mechanism is not devised to deliver e-care to uninsured Arab immigrant subgroups (Cowan et al., 2019).

The proposed policy solution, “increase the cultural competency of treatment providers,” can even situate the issue of mental health underrepresentation among Arab immigrants in its historical and cultural context, therefore drawing public attention to the issue as a social problem. Hilgartner and Bosk assert that “carrying capabilities exist at both the institutional and individual levels” (1988, p. 59). The public lacks the time and resources to care about every issue that is brought to their attention. Individual bearing capacities are “socially organized” (1988, p. 59), and hence, individuals may not care about issues that do not concern them. The issue of Arabs’ lack of access to mental health care is not pertinent to all individuals. Immigrants of diverse racial or cultural backgrounds or those without mental health issues may be unable of empathetic response or interest in matters unrelated to their immediate needs. Moreover, the notion of innovation offered in the public arena model was reflected in the two recommended policy solutions; mental health care integration and novel mode of mental health care endorsement, which stimulate public discourse around the growing incidence of mental health disorders among the Arab minority.

## Conclusion

There is evidence that Arab immigrants remain underrepresented in the mental health care system, despite their need for such treatments (Derr, 2016). However, this underrepresentation is not recognized as a public or policy concern (Khan et al., 2019). The Public Arenas Model serve as an effective tool for comprehending how Arabs’ access to mental health treatments has not been identified as a societal problem and how operators battle for attention and resources across public arenas to push their definitions of the problem. The application of Hilgartner and Bosk’s (1988) Model clarified previous definitions of the Arab minority’s underutilization of mental care promulgated within the health, policy, and cultural arenas (Alkaid et al., 2021; Suleiman et al., 2021). The use of the Model has also aided understanding of why mental health inaccessibility among Arabs is viewed as a less significant social problem unworthy of the attention paid to other issues (Smith, 2009). Several rudimentary problems related to Arab minorities’ access to mental health care are clarified after applying the Public Arena Model: (1) how the Arab minority’s underutilization of mental care was previously defined inaccurately as an issue of cultural and language incompetence instead of the more accurate assessment, i.e., a lack of access due to economic burden and the cost of mental care (Alkaid et al., 2021; Suleiman et al., 2021); (2) why mental health inaccessibility among Arabs is viewed as a less significant social problem not worth the attention paid to other social challenges; and, (3) how the efforts of many stakeholders to advocate, inform, and influence the public’s attention to mental health care for the Arab immigrants has strengthened this issue as a prominent topic of public debate (Smith, 2009).

Additionally, the use of the Model’s features contributes to the increasing traction of mental health service inaccessibility. Hilgartner and Bosk’s (1988) discussion of their Model suggested how drama and novelty could be employed to focus the public’s attention on and stimulate public discourse about the growing rates of mental health care needs among the Arab minority, as well as selection criteria enable the recognition of Arab mental health care as a social priority (Hilgartner & Bosk’s, 1988). We have proposed some policy options to address the issues surrounding access to mental health care for Arab immigrants. By adopting this conceptual framework, mental health practitioners, health policy experts, and activists may actively improve individual and minority community prospects (Khan et al., 2019).
